# Comparison of Conventional Open Thyroidectomy and Endoscopic Thyroidectomy via Breast Approach for Papillary Thyroid Carcinoma

**DOI:** 10.1155/2015/239610

**Published:** 2015-08-26

**Authors:** Zhuo Tan, JiaLei Gu, QianBo Han, WenDong Wang, KeJing Wang, MingHua Ge, JinBiao Shang

**Affiliations:** ^1^Department of Head and Neck Surgery, Zhejiang Cancer Hospital, Hangzhou 310022, China; ^2^Wenzhou Medical University, Wenzhou 325035, China

## Abstract

*Purpose*. The aim of this study was to evaluate the feasibility of endoscopic thyroidectomy via breast approach for papillary thyroid carcinoma (PTC). *Methods*. Between March 2008 and March 2013, 34 patients with PTC received endoscopic thyroidectomy (endo group) and 30 patients received conventional open thyroidectomy (open group). Patients in two groups underwent ipsilateral central compartment node dissection. The two groups were compared in terms of patient characteristics, perioperative clinical results, and postoperative complication. *Results*. The rates of lymph node metastasis in endo group and open group were 23.5% (8/34) and 13.3% (4/30), respectively, without statistically significant difference (*P* = 0.351). The mean number of lymph nodes dissected was 2.4 ± 2.9 in endoscopic group and 2.2 ± 1.9 in open group (*P* = 0.774). During the follow-up period, there was no recurrence or metastatic patients in two groups. All patients received the excellent cosmetic results in endo group, while 25 patients were satisfied with the cosmetic result and 5 were unsatisfied in the open group. *Conclusions*. The efficacy of endoscopic thyroidectomy via breast approach could be comparable to conventional open thyroidectomy in selected patients with PTC.

## 1. Introduction

Recently, with the development of endoscopic equipment and technology, thyroid surgery has evolved towards minimal incisions and endoscopic approaches. Endoscopic surgery can provide better functional and cosmetic results and rapid wound healing. At present, the endoscopic surgery is appropriate for thyroid benign lesions, and its application for thyroid carcinoma remains controversial. Since the attempt to perform endoscopic surgery on patients with papillary thyroid carcinoma (PTC) by Miccoli et al. in 2002 [1], several reports about endoscopic thyroidectomy for patients with low-risk thyroid carcinoma have emerged. Indications for endoscopic thyroidectomy in well-differentiated thyroid carcinoma included age < 45 years, tumor size < 2 cm, and no evidence of lymph node or local invasion [2]. The aim of this study was to evaluate the effects and cosmetic results of endoscopic thyroidectomy via breast approach for PTC, compared with conventional open thyroidectomy.

## 2. Materials and Methods

### 2.1. Patients

Between March 2008 and March 2013, a total of 34 patients with PTC underwent endoscopic thyroidectomy via breast approach in department of head and neck surgery of Zhejiang Cancer Hospital. And 30 patients underwent conventional open thyroidectomy. Both of patients in two groups underwent prophylactic ipsilateral central compartment node dissection. All patients received surgery by the same surgeon. There was no statistical significant difference of general data between two groups such as gender, anamnesis, and state of illness (*P* > 0.05).

B-ultrasound and CT scans of the neck were performed before the surgery and fine needle aspiration (FNA) was not routinely performed. Vocal cord movement was examined with laryngoscopy before and after the surgery. The eligibility criteria for endoscopic thyroidectomy were unilateral thyroid with single mass (diameter less than 2 cm), no extrathyroidal invasion; no metastasis to lateral cervical lymph nodes or distant metastasis, and patients with high expectation of cosmetic results. All patients in endoscopic group provided informed consent for endoscopic thyroidectomy and the possibility of conversion to open thyroidectomy.

We compared the operating time, perioperative bleeding, number of dissected lymph nodes, number of positive lymph nodes, rates of lymph node metastasis, amount of drainage, time of removing drainage tube, postoperative complications, and cosmetic results between endoscopic and open groups.

### 2.2. Operation Method

**Figure 1:**
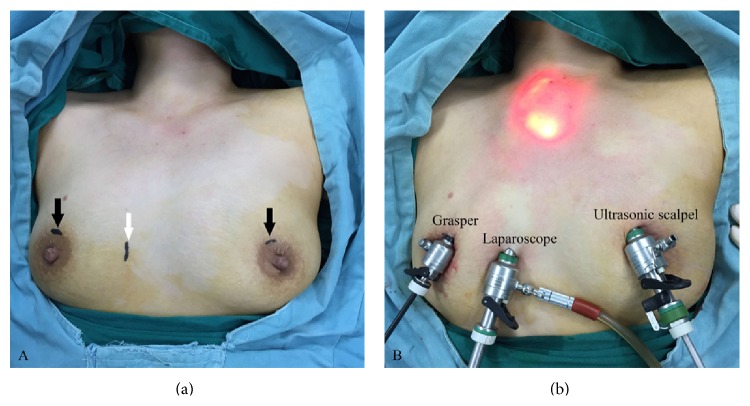
(a) Two incisions for the main and auxiliary operation ports (black arrow); the incision for laparoscope (white arrow); (b) external view after positioning the trocars.

Endoscopic procedure was as follows. (1) Position and anesthesia: patients were placed in a supine position while under general anesthesia. The neck was slightly extended. (2) Establishing subcutaneous working space: the designed working space was injected with 200–350 mL of “inflation liquid” (consisting of 1 mg adrenaline mixed with 500 mL saline) in the subcutaneous space. A 10 mm straight incision was made outside 2 cm of sternal partial lateral at the level of nipple, which could reduce the scar compared with the sternal median incision. A subcutaneous separation stick was used to separate the skin through the incision resulting in fanning out from the deep layer of the superficial fascia toward the suprasternal fossa for building an observation tube (trocar) tunnel for endoscope. Each of 5 mm incisions was made at the bilateral areola to establish the main and auxiliary operation ports for ultrasonic scalpel and grasper (Figures 1(a) and 1(b)). Then the subcutaneous loose connective tissue was separated directly by ultrasonic scalpel. (3) Separating and resecting thyroid tissue: cervical linea alba was dissected and the thyroid anterior muscles were separated by ultrasonic scalpel, and then the anterior cervical muscle group was retracted with suture to expose the thyroid tissue. The lower pole of thyroid was exposed through blunt dissection, and then the inferior thyroid arteries and veins were resected. The lobe was retracted superiorly and medially, and the middle thyroid vein and Berry's ligament were cut with ultrasonic scalpel. The recurrent laryngeal nerve (RLN) was exposed by blunt dissection to reveal its position and course. Subsequently, the superior thyroid arteries were exposed and resected after retracting the lobe inferiorly. Finally, the lobe was dissected from the trachea. (4) The resected specimens were sent for frozen section; if confirmed to be PTC, ipsilateral central compartment node dissection was performed routinely.

Conventional open procedure was as follows. A 4.0~5.5 cm curved collar skin incision was made in the mid-line of the anterior neck 2 cm above the sternal notch. Routine ipsilateral thyroidectomy plus isthmus resection were performed in the same manner as the endoscopic procedure, and specimens were sent for frozen section; if proved to be PTC, prophylactic central compartment node dissection was performed.

### 2.3. Statistical Analysis

All statistical analyses were carried out by SPSS (SPSS version 18.0, Chicago, IL). All data were expressed as mean ± SD, proportions, or numbers. Continuous data were compared using *t*-tests, and categorical data were analyzed using chi-square tests. A *P* value < 0.05 was considered statistically significant.

## 3. Results

The clinical characteristics of the two groups are shown in Table 1. 34 patients with PTC underwent endoscopic thyroidectomy (endo group) and 30 patients underwent conventional open thyroidectomy (open group). The median age of the patients was 30 years (range: 16–44 years) in the endo group and 43 years (range: 25–76 years) in the open group. The male-to-female gender ratios were 2 : 32 (1 : 16) in the endo group and 4 : 26 (1 : 6.5) in the open group. Unilateral thyroidectomy and prophylactic ipsilateral central compartment node dissection were performed for patients in both groups. There was no case of conversion to the open surgery in the endo group. The total operation time in the endo group was significantly longer than in the open group (95 ± 15 min versus 33 ± 5 min, *P* < 0.01). The mean number of the dissected lymph nodes was 2.4 ± 2.9 in the endo group and 2.2 ± 1.9 in the open group (*P* = 0.774). The mean number of positive lymph nodes was 0.8 ± 2.0 in the endo group and 0.2 ± 0.7 in the open group (*P* = 0.114). The mean volume of intraoperative bleeding in endo group was more than in open group (30 ± 37 mL versus 16 ± 10 mL, *P* = 0.039). The mean amount of postoperative drainage in the endo group was more than in open group (166 ± 75 mL versus 57 ± 30 mL, *P* < 0.01), and the time of removing drainage tube in the endo group was longer than in open group (4.8 ± 1.4 d versus 2.7 ± 0.7 d, *P* < 0.01). There was no significant difference in tumor size between the two groups (0.7 ± 0.3 cm versus 0.8 ± 0.4 cm, *P* = 0.121). The incidence of capsular invasion was 5.9% (2/34) in endo group and 10.0% (3/30) in open group (*P* = 0.884). All cases of capsular invasion were revealed by postoperative pathological examination, not shown by intraoperative frozen section and preoperative examination. All patients received the excellent cosmetic results in endo group, while 25 patients were satisfied with the cosmetic result and 5 were unsatisfied in the open group.

The postoperative complications in the two groups are shown in Table 2. Of the 34 patients in endo group, 5 cases suffered from subcutaneous emphysema after surgery, which may be related to the establishment of endoscopic channel, and it restored after conservative treatment. One patient in endo group experienced transient hoarseness and recovered after two months of conservative treatment. Tracheal fistula occurred in 1 patient in endo group, and the patient recovered after open operation repair. Postoperative temporary hypocalcaemia was observed in one patient in the open group, with recovery after calcium supplements. Postoperative subcutaneous emphysema (5 cases) and ecchymosis (15 cases) occurred in endo group. There was no tumor recurrence or metastasis in both groups during the follow-up period.

## 4. Discussion

The incidence of thyroid cancer, especially PTC, is on the rise. Thyroid cancer was the sixth most common cancer for women in America in 2009 [3]. As the rapidly increasing incidence of thyroid carcinoma in young women, cosmetic result plays an important role in thyroid surgery. Total endoscopic thyroidectomy (TET) is mainly performed for benign thyroid disease because of its advantages of leaving no scars in the neck. It has been widely accepted, especially by young women with thyroid tumor. But how to deal with the patients with PTC confirmed by frozen section biopsy remains controversial. In the early stage of TET, thyroid carcinoma was considered as the contraindication of TET [4, 5], and most scholars advocated that it should be immediately transferred to open surgery for completed resection [6, 7]. However, Miccoli et al. reported that 12 patients with PTC successfully underwent endoscopic thyroidectomy in 2001 [8], and subsequent other studies also published the similar reports [9]. With the improvement of endoscopic technique and surgeon's experience, more and more researchers recognized that endoscopic surgery for PTC could receive the radical cure result with strictly grasping the indications. In fact, the debate lies in the feasibility of radical cure through endoscopic surgery for PTC. Our research showed that the mean number of dissected lymph nodes was 2.4 ± 2.9 in endo group and 2.2 ± 1.9 in the open group, without significant difference (*P* = 0.774). The rate of lymph node metastasis in two groups was 23.5% (8/34) and 13.3% (4/30), respectively, and there was also no statistically significant difference (*P* = 0.351). During the follow-up period, there was no recurrence or metastatic cases in both groups. Patients in endoscopic group were more satisfied with cosmetic results than those in the open group. According to our results, patients (T1, CN0) with PTC who received endoscopic thyroidectomy could achieve similar efficacy, compared with those who received conventional open thyroidectomy. Meanwhile, patients could obtain more cosmetic satisfaction without noticeable cervical scar by endoscopic surgery. Our results demonstrated that immediate conversion to open operation was not necessary when the specimen was confirmed malignant during endoscopic surgery.

With the improved visualization by endoscope, the recurrent laryngeal nerve was exposed to reveal its position and course distinctly and conveniently by skilled surgeons, but the thermal damage of ultrasonic scalpel maybe injured the RLN. Owaki et al. suggested that there was no damage to the nerve histologically when the ultrasonic coagulating and cutting systems were used for less than 20 seconds at a distance of 3 mm to RLN [10]. We summarized that functional knife head should be kept away from the nerve and burning time should be reduced as soon as possible while operating near the RLN. After using ultrasonic scalpel, the cutter head should be cooled before the next step, in order to avoid the heat damage to the nerve. Hoarseness occurred in 1 case in endo group, and this patient resumed with the normal movement of vocal cord shown by laryngoscopy after 2 months. 1 patient in endo group suffered from tracheal leak, which was caused by the intraoperative heat damage of ultrasonic scalpel. The adequate spacing distance between ultrasonic scalpel and important tissues was essential to reduce postoperative complication. It is important to identify the boundary and extent of central compartment node dissection for reserving inferior parathyroid glands during thyroid surgery. With the amplification of endoscopy, parathyroid glands show pale orange red on the screen, while adipose tissues show yellow. And it is relatively easy to recognize them by the obvious different colors. Although the parathyroid gland can be preserved, the blood supply may be affected by the thermal damage of ultrasound knife, especially inferior parathyroid gland after the lymph node dissection. The lower bound of central compartment node dissection was generally defined as the superior border of manubrium of sternum. Due to the barrier of the sternum and clavicle, there was a certain blind area during endoscopic lymph node dissection. Therefore, patients should receive CT scan and ultrasound to exclude enlarged mediastinal or supraclavicular lymph nodes before endoscopic surgery. Although several studies have reported the successful cases of endoscopic lateral neck lymph node dissection [11], the cervical lateral lymph node dissection is not advocated in endoscopic surgery due to the inadequate exposure.

The operation time, the amount of fluid drainage, and the time of removing drainage tube in endoscopic group were much more than those in the open group. A larger working space was made by separating skin flap from the chest wall to neck during endoscopic operation, which resulted in more exudates after surgery. Whether endoscopic thyroidectomy belongs to minimally invasive operation remains controversial [12]. The subcutaneous wound of chest wall is larger in endoscopic thyroidectomy than cervical wound in conventional operation. So we regard it as the cosmetic surgery rather than minimally invasive operation. With the continuous improvement of laparoscopic equipment and operation skill, endoscopic thyroidectomy for PTC will be commonly accepted.

At present it has been reported that subcutaneous tumor implantation of thyroid and chest occurs in patients with endoscopic thyroidectomy [13]. Therefore, it is necessary to resect tumor completely and take it out with specimen collection bag wrapping during the operation. At the end of endoscopic operation, the wounds should be repeatedly washed with a large amount of distilled water in order to reduce the implantation of chest wall.

As described above, endoscopic thyroidectomy is an effective alternative for selected patients with PTC compared with conventional open thyroidectomy. The major limitations in this study were the short follow-up and the shortage of patients. But the surgical outcomes of endoscopic surgery were favorable compared with conventional open thyroidectomy. In the future, it is necessary to accumulate more cases and longer follow-up to determine the curative effect.

## 5. Conclusions

In summary, our results suggest that endoscopic thyroidectomy is comparable to conventional open thyroidectomy when performed in properly selected patients with PTC by experienced endoscopic thyroid surgeons.

## Figures and Tables

**Table 1:** Comparison of clinical characteristics of the patients in two groups.

	Endoscopic (*n* = 34)	Open (*n* = 30)	*P* value
Gender			0.555
Female	32 (94.1%)	26 (86.7%)	
Male	2 (5.9%)	4 (13.3%)	
Median age (range, years)	30 (16–44)	43 (25–76)	<0.05
Mean tumor size (cm)	0.7 ± 0.3	0.8 ± 0.4	0.121
Capsular invasion	2 (5.9%)	3 (10.0%)	0.884
Lymph node metastasis	8 (23.5%)	4 (13.3%)	0.351
Operation time (min)	95 ± 15	33 ± 5	<0.01
Intraoperative bleeding (mL)	30 ± 37	16 ± 10	0.039
Number of dissected lymph nodes	2.4 ± 2.9	2.2 ± 1.9	0.774
Number of positive lymph nodes	0.8 ± 2.0	0.2 ± 0.7	0.114
Time of removing drainage (days)	4.8 ± 1.4	2.7 ± 0.7	<0.01

**Table 2:** Comparison of complications in endoscopic and open groups.

Complications	Endoscopic (*n* = 34)	Open (*n* = 30)
Temporary hypocalcaemia	0	1 (3.3%)
Transient RLN palsy	1 (2.9%)	0
Bleeding	0	0
Asphyxia	0	0
Superior laryngeal nerve injury	0	0
Subcutaneous emphysema	5 (14.7%)	0
Subcutaneous ecchymosis	15 (44.1%)	0
Tracheal fistula	1 (2.9%)	0

## References

[1] Miccoli P., Elisei R., Materazzi G. (2002). Minimally invasive video-assisted thyroidectomy for papillary carcinoma: a prospective study of its completeness. *Surgery*.

[2] Kitano H., Fujimura M., Kinoshita T., Kataoka H., Hirano M., Kitajima K. (2002). Endoscopic thyroid resection using cutaneous elevation in lieu of insufflation: technical considerations and review of an open series. *Surgical Endoscopy and Other Interventional Techniques*.

[3] Jemal A., Siegel R., Ward E., Hao Y., Xu J., Thun M. J. (2009). Cancer statistics, 2009. *CA: Cancer Journal for Clinicians*.

[4] Gagner M. (1996). Endoscopic subtotal parathyroidectomy in patients with primary hyperparathyroidism. *The British Journal of Surgery*.

[5] Hüscher C. S., Chiodini S., Napolitano C., Recher A. (1997). Endoscopic right thyroid lobectomy. *Surgical endoscopy*.

[6] Park Y. L., Han W. K., Bae W. G. (2003). 100 Cases of endoscopic thyroidectomy: breast approach. *Surgical Laparoscopy, Endoscopy & Percutaneous Techniques*.

[7] Choe J.-H., Kim S. W., Chung K.-W. (2007). Endoscopic thyroidectomy using a new bilateral axillo-breast approach. *World Journal of Surgery*.

[8] Miccoli P., Berti P., Raffaelli M., Conte M., Materazzi G., Galleri D. (2001). Minimally invasive video-assisted thyroidectomy. *American Journal of Surgery*.

[9] Lombardi C. P., Raffaelli M., Princi P., De Crea C., Bellantone R. (2007). Minimally invasive video-assisted functional lateral neck dissection for metastatic papillary thyroid carcinoma. *American Journal of Surgery*.

[10] Owaki T., Nakano S., Arimura K., Aikou T. (2002). The ultrasonic coagulating and cutting system injures nerve function. *Endoscopy*.

[11] Li Z., Wang P., Wang Y. (2011). Endoscopic lateral neck dissection via breast approach for papillary thyroid carcinoma: a preliminary report. *Surgical Endoscopy and Other Interventional Techniques*.

[12] Tan C. T. K., Cheah W. K., Delbridge L. (2008). “Scarless” (in the neck) endoscopic thyroidectomy (SET): an evidence-based review of published techniques. *World Journal of Surgery*.

[13] Kim J. H., Choi Y. J., Kim J. A. (2008). Thyroid cancer that developed around the operative bed and subcutaneous tunnel after endoscopic thyroidectomy via a breast approach. *Surgical Laparoscopy, Endoscopy & Percutaneous Techniques*.

